# Academic Stress in Doctoral Research Scholars in Relation to Biochemical Markers and Mental Health Symptoms: A Single-Center Cross-Sectional Study

**DOI:** 10.3390/healthcare14193280

**Published:** 2026-10-02

**Authors:** Kashif Abbas, Nazura Usmani, Safia Habib, Mudassir Alam, Sahab Kausar

**Affiliations:** 1 Department of Zoology, Faculty of Life Sciences, Aligarh Muslim University, Aligarh 202001, India; kashifabbas68@yahoo.com; 2 Department of Biochemistry, Faculty of Medicine, J.N. Medical College, Aligarh Muslim University, Aligarh 202001, India; 3 Department of Biological Sciences, Indian Biological Sciences and Research Institute (IBRI), Noida 201301, India; syedalamalig@gmail.com; 4 Department of Statistics and Operations Research, Aligarh Muslim University, Aligarh 202001, India

**Keywords:** academic stress, doctoral scholars, HbA1c, cortisol, C-reactive protein, sleep quality

## Abstract

**Background:** Doctoral training involves prolonged performance demands, uncertainty, supervisory dependence, and career pressure, yet objective biochemical correlates of academic stress remain incompletely characterized. This study examined academic stress in relation to glycemic, neuroendocrine, inflammatory, and thyroid-related markers, alongside psychological symptoms and sleep quality, in doctoral research scholars. **Methods:** This cross-sectional study included 203 doctoral research scholars (123 female, 80 male; mean age 27.2 years). Academic stress was assessed using a 34-item doctoral-adapted Academic Stress Scale (AST). Glycated hemoglobin (HbA1c), evening cortisol, C-reactive protein (CRP), and thyroid-stimulating hormone (TSH) were measured as biochemical outcomes. Depression, anxiety, and stress symptoms were assessed using the 21-item Depression Anxiety Stress Scales (DASS-21), and sleep quality using the Pittsburgh Sleep Quality Index (PSQI). Primary associations were examined using linear regression with heteroscedasticity-consistent type 3 (HC3) robust standard errors, adjusted for age, gender, body mass index, and lifestyle activity, with false-discovery-rate (FDR) correction. **Results:** Mean AST was 109.5 (standard deviation 21.0). Above-normal DASS-21 scores were observed for depression in 64.5%, anxiety in 86.7%, and stress in 49.3%, while 79.3% had poor sleep quality. Higher AST was associated with all four biochemical markers. Per 10-point increase in AST, HbA1c was 0.225 percentage points higher (95% confidence interval 0.158–0.291), evening cortisol 0.502 µg/dL higher (0.299–0.706), CRP 13.9% higher (8.4–19.7%), and TSH 0.125 µIU/mL higher (0.049–0.200); all FDR *q* ≤ 0.0013. Depression and anxiety severity distributions differed by gender, whereas stress did not. Exploratory analyses showed positive correlations between DASS-21 symptoms and all four biomarkers (Spearman *ρ* = 0.22–0.58; all FDR *q* < 0.01). **Conclusions:** Higher academic stress was associated with variation across glycemic, neuroendocrine, inflammatory, and thyroid-related markers in doctoral scholars, alongside substantial psychological symptom and sleep burden. The cross-sectional design precludes conclusions regarding temporal direction or causality and supports the need for longitudinal research on doctoral health and well-being.

## 1. Introduction

Doctoral education is intellectually demanding and can involve sustained uncertainty, performance evaluation, publication pressure, financial and career insecurity, and dependence on supervisory and institutional structures. These demands extend over several years and often coincide with important developmental and occupational transitions in young adulthood. Doctoral mental health has therefore become an increasingly important educational and public-health concern [1]. A meta-analysis of graduate students estimated that approximately one-third experienced anxiety, with doctoral students showing a slightly higher pooled prevalence than master’s students [2]. Doctoral-specific studies have likewise documented substantial psychological symptom burden and identified academic, supervisory, and organizational conditions as relevant correlates [3,4]. Longitudinal administrative evidence from Sweden further indicates that psychiatric medication use increases after entry into PhD training and across the doctoral period before declining around the period in which most candidates graduate [5].

Academic stress in doctoral scholars differs from short-duration examination stress because it may arise from recurring research milestones, publication and evaluation cycles, time-management demands, peer comparison, collaborative pressures, and self-imposed performance standards. Studies of graduate and doctoral populations have linked these academic conditions with distress, poorer well-being, and impaired sleep [6,7]. However, much of this literature has relied on questionnaire-based outcomes, leaving less clear whether doctoral-specific academic stress is also accompanied by measurable biological variation in young scholars without reported chronic morbidity.

Stress-related physiological responses provide several plausible, non-exclusive pathways linking prolonged psychological demands with metabolic, neuroendocrine, inflammatory, and thyroid-related variation. The hypothalamic–pituitary–adrenal (HPA) axis regulates glucocorticoid responses to stress, making cortisol a relevant neuroendocrine marker, although a single serum measurement cannot characterize the full diurnal HPA profile [8]. Stress may also coincide with metabolic and inflammatory changes through endocrine and autonomic signaling, sleep disruption, behavioral factors, and immune regulation [9].

Within this framework, HbA1c provides an integrated measure of glycaemic exposure over preceding weeks, whereas CRP provides a nonspecific indicator of systemic inflammatory variation. Evidence linking psychosocial stress with HbA1c in younger non-clinical populations remains limited and inconsistent [10,11]. CRP has been associated with psychosocial exposures, although its nonspecific nature limits attribution to psychological stress. TSH provides a complementary marker of thyroid-axis variation; experimental evidence indicates that acute psychosocial stress can transiently alter TSH [12]. Thus, these four markers represent complementary physiological domains rather than specific biomarkers of psychological stress.

Despite evidence that doctoral training is associated with substantial psychological and sleep burden, limited research has examined doctoral-specific academic stress together with multiple objective biochemical markers within the same sample. Existing stress-biomarker literature has largely examined general or psychosocial stress exposures or individual physiological outcomes, making it difficult to determine whether a doctoral-specific stress construct shows a consistent pattern across multiple biological systems.

The present study therefore evaluated total academic stress in relation to HbA1c, evening serum cortisol, CRP, and TSH in a defined sample of doctoral research scholars, while characterizing psychological symptoms and sleep quality as contextual outcomes. The primary hypothesis was that higher total Academic Stress Scale (AST) scores would be associated with higher values of the four biochemical markers after adjustment for age, gender, body mass index (BMI), and lifestyle activity. Robustness and sensitivity analyses evaluated the stability of these primary associations under alternative statistical specifications. Secondary analyses examined gender-related patterns, whereas planned exploratory analyses examined potential gender modification of the AST–biomarker relationships, DASS-21–biomarker correlations, and individual academic-stress domains. Given the cross-sectional design, the study was designed to estimate associations and heterogeneity rather than causal mechanisms or mediation.

## 2. Methods

### 2.1. Study Design and Participants

A cross-sectional observational study was conducted among doctoral research scholars at Aligarh Muslim University in North India. Data were collected during September–October 2025. Eligible participants were currently enrolled doctoral research scholars who were able to complete the study questionnaires and provide a venous blood sample. Doctoral research scholars were approached individually within the study setting and invited to participate. Recruitment was voluntary and used a non-probability, convenience-based approach. Therefore, the recruitment strategy did not involve systematic invitation of all eligible doctoral research scholars at the institution.

A total of 238 individuals initially responded. Thirty-five were excluded because of incomplete questionnaire responses (*n* = 6), unwillingness to provide a blood sample (*n* = 14), self-reported thyroid disorders (*n* = 8), hypertension (*n* = 4), or use of sleep medications (*n* = 3), yielding a final analytic sample of 203 participants aged 24–33 years. All 203 participants had complete data for the variables used in the present analyses, and no statistical imputation was required. No formal a priori power calculation was specified in the study protocol; therefore, the achieved sample reflected eligible participants who completed the study procedures during the recruitment period. To characterize the statistical sensitivity of the achieved sample, a sensitivity power analysis was subsequently performed. For the primary multiple linear regression models with five predictors, a sample of 203 provided 80% power at α = 0.05 to detect a minimum overall effect size of Cohen’s *f*^2^ = 0.065 (approximately equivalent to *R*^2^ = 6.1%). For the AST × gender interaction models, which contained six predictors, the corresponding minimum detectable incremental interaction effect was Cohen’s *f*^2^ = 0.039 at α = 0.05 and 80% power. The interaction and other secondary or exploratory analyses were not used to determine the sample size and were interpreted accordingly, with false-discovery-rate correction applied within the predefined test families. The study was approved by the Institutional Ethics Committee (D.No. IECJNMC/1659; 4 September 2024), and all participants provided written informed consent before data collection.

### 2.2. Academic Stress Assessment

Academic stress was assessed using a doctoral-context adaptation of the 34-item Academic Stress Scale described by Lin and Chen (2009) [13]. The original instrument assesses academic stress across seven domains using a five-point Likert response format. For the present study, the 34-item format and five-point response structure were retained, while item wording and domain context were adapted where necessary to reflect the research-intensive structure and recurring demands of doctoral training. The adapted domains were Supervisor-Related Stress (SRS), Publication/Outcome Stress (RS), Evaluation Stress (TS), Collaboration Stress (SGS), Peer Comparison Stress (PS), Time Management Stress (TMS), and Self-Inflicted Stress (SIS). Undergraduate- and coursework-oriented references were reframed in corresponding doctoral-research contexts. Representative examples of the adapted wording included “My supervisor’s expectations for my research output cause me stress,” “Defending my research in front of peers or examiners is stressful,” and “Comparing my research progress with peers causes me stress.”

The adapted questionnaire was reviewed by both study supervisors for contextual relevance, clarity, and applicability to doctoral research before pilot administration. This review served as a contextual appraisal of the adapted wording rather than a formal psychometric validation procedure. The possible total score ranged from 34 to 170, with higher scores indicating greater academic stress. Participants were instructed to rate the items with reference to their experiences during doctoral training; thus, the AST reflected academic-stress burden within the doctoral-training context rather than stress over a fixed short-term recall interval.

A subset of 50 participants from the study sample underwent pilot assessment of the adapted questionnaire before the main analyses. The pilot assessment was undertaken to evaluate contextual applicability and internal consistency. Cronbach’s alpha was 0.930 for the total scale and 0.885 for SRS, 0.890 for RS, 0.866 TS, 0.774 for SGS, 0.859 for PS, 0.749 for TMS, and 0.783 for SIS (Supplementary Table S1). No further modifications to the questionnaire were made after the pilot assessment, and these 50 participants were retained in the final analytic sample because the same questionnaire version was used throughout the study.

No formal exploratory or confirmatory factor analysis was performed for the contextually adapted version. Accordingly, the present study does not claim independent construct validation of the adapted domain structure or present the instrument as a newly validated scale. The total AST score served as the primary exposure in analyses of the biochemical outcomes. Individual academic-stress domains were examined separately in relation to HbA1c and evening cortisol as planned exploratory analyses. Because the adapted domain structure was not formally re-evaluated for construct validity in the present sample, these domain-level findings were treated as preliminary evidence and were not interpreted as independent validation of the domain structure.

### 2.3. Psychological Distress and Sleep Quality

Depressive, anxiety, and stress symptoms were assessed with the original English version of the 21-item Depression Anxiety Stress Scales (DASS-21) [14,15]. The standardized questionnaire was administered in its original form without modification. The three seven-item subscales were scored according to conventional procedures, with subscale sums multiplied by two for comparison with established DASS severity ranges. Depression was classified as normal (0–9), mild (10–13), moderate (14–20), severe (21–27), or extremely severe (≥28); anxiety as normal (0–7), mild (8–9), moderate (10–14), severe (15–19), or extremely severe (≥20); and stress as normal (0–14), mild (15–18), moderate (19–25), severe (26–33), or extremely severe (≥34). These categories represent symptom-screening severity ranges and were not interpreted as clinical psychiatric diagnoses. In the present sample, the DASS-21 demonstrated good internal consistency, with a Cronbach’s alpha of 0.89.

Sleep quality was assessed with the original English version of the Pittsburgh Sleep Quality Index (PSQI) [16]. The standardized questionnaire was administered in its original form without modification. Higher scores indicate poorer sleep quality, and a global score > 5 was used to identify poor sleep for descriptive prevalence estimates. In the present sample, the PSQI demonstrated acceptable internal consistency, with a Cronbach’s alpha of 0.73. DASS-21 and PSQI measures were treated primarily as behavioral and mental-health context rather than as presumed mediators of biochemical outcomes.

### 2.4. Demographic, Anthropometric, and Lifestyle Variables

Age, self-reported gender, urban/rural demography, socioeconomic category, and lifestyle activity were recorded by a study performa. Lifestyle was classified as sedentary or active from self-reported regular physical exercise and daily inactivity: participants reporting no regular physical exercise and approximately eight hours of daily inactivity were categorized as sedentary, whereas those reporting regular physical exercise, such as gym-based workouts or participation in sports, on at least five days per week and lower daily inactivity were categorized as active. This was a study-specific, coarse behavioral classification based on exercise frequency and habitual daily inactivity; exercise duration and intensity were not separately quantified. Because this was a coarse behavioral classification rather than a validated physical-activity instrument, it was used as an adjustment variable and interpreted conservatively. Socioeconomic status was not included as a primary model covariate. Socioeconomic status was assessed using self-reported monthly family income as an income-based socioeconomic indicator and categorized as lower-income (<₹24,750/month), middle-income (₹24,750–₹65,999/month), or upper-income (≥₹66,000/month). These categories were used as a broad income-based characterization rather than as a formal multidimensional socioeconomic scale. Body weight and height were measured using standard procedures, and BMI was calculated as weight in kilograms divided by height in meters squared. Participants also reported the timing of their first and last daily meals. A self-reported daily eating-window duration was calculated as the interval between these times and used only in sensitivity analyses of HbA1c. It was not interpreted as adherence to time-restricted eating.

### 2.5. Blood Collection and Biochemical Outcome Assessment

Venous blood was collected from each participant in the evening at approximately 18:00. A total of 5 mL was obtained under aseptic conditions. Approximately 1 mL was transferred to an EDTA-anticoagulated tube for HbA1c measurement, and the remaining 4 mL was collected for serum-based assays. Serum was separated immediately after blood collection by centrifugation at approximately 522× *g* for 15 min (2000 rpm) using a REMI C-854/8 centrifuge. The serum was transferred into labeled tubes and refrigerated at 4 °C until analysis the following day. The concurrently collected EDTA whole-blood specimen for HbA1c was likewise stored at 4 °C and analyzed the following day.

Serum TSH was measured using a third-generation chemiluminescent immunoassay on the Beckman Coulter UniCel DxI 800 immunoassay system (Beckman Coulter, Inc., Brea, CA, USA). The method uses a paramagnetic particle-based two-site sandwich immunoenzymatic format and is standardized to the WHO Third International Standard for human TSH [17,18]. The manufacturer-specified limit of detection is ≤0.005 µIU/mL and the limit of quantitation is ≤0.01 µIU/mL. Manufacturer performance data report total imprecision of approximately 3.1–6.3% CV across evaluated TSH concentrations, with a specified total imprecision of ≤10% CV at concentrations > 0.02 µIU/mL. An active calibration curve is required, with manufacturer-specified calibration every 28 days using the corresponding Access TSH calibrators.

Serum cortisol was quantified using the Access Cortisol assay (Beckman Coulter, Ref. 33600) on the UniCel DxI 800 Access Immunoassay System (Beckman Coulter, Inc., Brea, CA, USA). This paramagnetic particle-based competitive chemiluminescent immunoenzymatic assay measures endogenous cortisol through competition with enzyme-labeled cortisol for antibody-binding sites; emitted chemiluminescence is inversely proportional to cortisol concentration [19]. The manufacturer-reported analytical sensitivity is 0.4 µg/dL, with an analytical measuring range of approximately 0.4–60.0 µg/dL. Manufacturer-reported total imprecision is <12% at approximately 5 µg/dL and <10% at higher concentrations; representative serum-control CVs were 7.9%, 6.0%, and 6.4% at mean concentrations of approximately 6.0, 24.1, and 38.4 µg/dL, respectively. Calibration is based on a stored multi-point curve and is required every 28 days according to the manufacturer’s instructions. Because cortisol follows a pronounced diurnal rhythm, results are described specifically as evening serum cortisol rather than as a measure of total daily cortisol output.

HbA1c was measured in whole blood using the Premier Hb9210 analyzer (Trinity Biotech, Bray, Ireland), an automaated high-performance liquid chromatography system based on boronate-affinity chromatography [20]. Glycated and non-glycated haemoglobin fractions were separated and quantified photometrically by the instrument. Results were expressed as HbA1c percentage under National Glycohemoglobin Standardization Program standardization traceable to the Diabetes Control and Complications Trial reference framework [21]. Calibration and result standardization followed the manufacturer’s IFCC/NGSP traceability framework. In a published multicentre evaluation of the Premier Hb9210, mean total CVs in NGSP percentage units were 1.62%, 1.59%, and 1.68% at low, medium, and high HbA1c concentrations, respectively [20].

Serum CRP was measured using the high-sensitivity application of a fully automated latex-enhanced immunoturbidimetric assay on a Beckman Coulter AU5821 analyzer (Beckman Coulter, Inc., Brea, CA, USA). Serum CRP reacts with anti-human CRP-coated latex particles, and the resulting increase in turbidity is measured photometrically at 570 nm. For the manufacturer-specified high-sensitivity application on the AU5800-series platform, the analytical measuring range is 0.2–80 mg/L, with concentrations below 0.2 mg/L falling below the stated measuring range. The manufacturer specifies within-run and total precision of SD ≤ 0.02 mg/L for the high-sensitivity application at low concentrations. The sample median CRP concentration of 1.35 mg/L was therefore well within the analytical measuring range and above its lower analytical limit, supporting adequate analytical sensitivity for evaluating low-grade CRP variation in this analytic sample. Calibration used the corresponding Beckman Coulter CRP Latex Highly Sensitive calibrator set and followed the manufacturer’s specifications [22].

Manufacturer-reported analytical performance characteristics and calibration procedures were available in the respective assay documentation as cited above. These values represent manufacturer-specified analytical performance rather than laboratory-derived study-specific estimates. Laboratory-specific internal quality-control records and study-period intra- and inter-assay CV estimates were not available to the research team and therefore are not reported.

### 2.6. Descriptive Concentration Categories

To facilitate descriptive interpretation of the biomarker distributions, concentration categories were defined using published reference ranges and commonly used interpretive thresholds, with these ranges further subdivided into descriptive bands where appropriate. These categories were intended to contextualize the observed concentrations within the study sample and were not used to establish clinical diagnoses or to imply that all category boundaries represented laboratory-specific diagnostic cut-offs.

Evening serum cortisol concentrations were categorized as low-range (<3 µg/dL), reference-range (3.0–7.0 µg/dL), upper reference-range (>7.0–10.0 µg/dL), and above the cited upper evening threshold (>10.0 µg/dL) [23,24]. The >10 µg/dL threshold was used as the upper boundary for descriptive interpretation of evening cortisol, while the lower range was further subdivided to characterize the distribution of evening measurements. Given the pronounced diurnal variation in cortisol concentrations, these categories were interpreted specifically in relation to the evening sampling time used in the present study. Values > 10 µg/dL were therefore regarded as above the cited evening reference threshold but were not interpreted as diagnostic evidence of hypercortisolism or other endocrine disease. Manufacturer documentation for the Access Cortisol assay likewise notes that single-serum PM cortisol concentrations reported in the literature are generally <10 µg/dL.

TSH concentrations were categorized as below the commonly cited reference interval (<0.4 µIU/mL), lower reference-range (0.4–2.5 µIU/mL), upper reference-range (>2.5–4.0 µIU/mL), and above the commonly cited upper reference limit (>4.0 µIU/mL) [25,26]. The 0.4–4.0 µIU/mL interval represents a commonly used adult reference range in the cited thyroid literature, and this interval was subdivided at 2.5 µIU/mL to characterize the upper portion of the distribution. Values > 4.0 µIU/mL were therefore interpreted as being above a commonly cited upper reference limit, but not as diagnostic evidence of thyroid dysfunction.

CRP concentrations were categorized as low-range (<1 mg/L), upper low-range (1–3 mg/L), minor/low-grade inflammatory elevation (>3–10 mg/L), moderate inflammatory elevation (>10–100 mg/L), and marked inflammatory elevation (>100 mg/L) [27]. The <1 and 1–3 mg/L bands represented descriptive subdivisions within the lower CRP concentration range, whereas the >3–10, >10–100, and >100 mg/L bands corresponded to progressively greater CRP elevations associated with increasing inflammatory activity in the literature. CRP concentrations between 3 and 10 mg/L have been described as minor elevations associated with low-grade inflammation, while higher concentrations are associated with progressively greater inflammatory responses [28]. Because CRP is a nonspecific acute-phase reactant, these categories were used to provide descriptive inflammatory context and were not interpreted as diagnostic evidence of a specific inflammatory disease or as universally applicable clinical severity grades.

HbA1c was classified as normal (<5.7%), prediabetes (5.7–6.4%) and diabetes range (≥6.5%) according to American Diabetes Association criteria [29,30].

### 2.7. Statistical Analysis

Statistical analyses were conducted in Python 3.13.5 within Jupyter Notebook 7.5.3. For the final reproducibility run, the analytical workflow was re-executed using Python 3.13.5, pandas 2.2.3, NumPy 2.3.5, SciPy 1.17.0, statsmodels 0.14.6, Matplotlib 3.10.8, and openpyxl 3.1.5, reproducing the reported core statistical results [31,32]. Continuous variables were summarized using mean and standard deviation or median and interquartile range, as appropriate, and categorical variables using counts and percentages. All tests were two-sided with a nominal α = 0.05. Benjamini–Hochberg false-discovery-rate (FDR) correction was applied within predefined families of related comparisons.

The analytical hierarchy was defined a priori. Associations of total Academic Stress Scale (AST) score with HbA1c, evening serum cortisol, CRP, and TSH constituted the primary analyses; gender-related comparisons were treated as secondary analyses; and DASS–biomarker correlations, AST-by-gender interactions, and academic-stress-domain analyses were planned exploratory analyses. The study and statistical analysis plan were not formally preregistered in a public registry.

For the primary analyses, unadjusted AST–biomarker associations were assessed using Pearson correlation coefficients with 95% confidence intervals. Spearman correlations using the raw biomarker values were examined as a nonparametric sensitivity analysis. CRP was natural-log transformed for correlation and regression analyses because of its right-skewed distribution. Separate ordinary least-squares regression models were fitted for each biochemical outcome and adjusted a priori for age, gender, BMI, and lifestyle activity, selected on substantive grounds as plausible correlates of stress or biochemical variation rather than according to statistical significance. HC3 heteroscedasticity-robust standard errors and 95% confidence intervals were used for the primary regression models. Associations were reported as unstandardized effects per 10-point higher AST, standardized *β* coefficients, and adjusted *R*^2^. For ln(CRP), coefficients were exponentiated to express the estimated percentage difference in CRP per 10-point higher AST. FDR correction was applied across the four primary biomarker tests. Full covariate estimates are provided in Supplementary Table S2.

Model diagnostics assessed residual distribution, heteroscedasticity, functional form, multicollinearity, and influential observations. Quadratic AST terms were examined for evidence of material nonlinearity, and no observation was automatically excluded on the basis of an outlier or influence statistic. Robust M-estimation was used as an influence-resistant sensitivity analysis; ln(TSH) was used for the TSH robust-regression model. Outcome-specific sensitivity analyses included exclusion of HbA1c values < 3.0%, natural-log transformation of TSH, Gamma regression with a log link for CRP, and additional adjustment for urban/rural demography, PSQI, and self-reported eating-window duration where conceptually relevant. Following the cortisol model-specification diagnostic, additional checks examined simultaneous quadratic terms for AST, age, and BMI and an ln(cortisol) sensitivity model. These analyses were treated as robustness checks rather than alternative primary models.

Gender-related analyses were treated as secondary analyses. Continuous differences between females and males were examined using Welch independent-samples comparisons with Hedges’ *g*, with Mann–Whitney tests used as distribution-robust sensitivity analyses. Binary categorical differences were summarized using prevalence ratios and assessed with chi-square or Fisher’s exact tests as appropriate. Differences in five-level DASS-21 severity distributions were examined using chi-square or permutation procedures for sparse tables, with Cramer’s *V* used as an effect-size measure. Detailed gender-comparison results are provided in Supplementary Tables S3A,B and S4.

Planned supplementary analyses examined Spearman correlations between DASS-21 subscales and the four biomarkers, with 95% confidence intervals obtained from 5000 bootstrap resamples and FDR correction across the 12-test family (Supplementary Table S5). As a supplementary robustness check, a DASS-21 total symptom score was calculated by summing the conventionally scored depression, anxiety, and stress subscales and correlated with each biomarker using the same Spearman-bootstrap procedure. FDR correction was applied across the four total-score–biomarker comparisons. This composite was used only to characterize overall symptom burden and was not interpreted as an independent diagnostic construct. Potential gender modification of the AST–biomarker associations was evaluated by adding centered AST-by-gender interaction terms to the adjusted models, with FDR correction across the four interaction tests; statistically supported interaction findings were further assessed using robust regression (Supplementary Table S6). The study sample was not primarily designed for interaction testing, and potentially relevant sex-related endocrine factors, including menstrual phase, reproductive hormone status, and hormonal contraceptive use, were not systematically characterized.

Finally, academic-stress domains were examined separately for HbA1c and evening cortisol using adjusted standardized models. Percentage-of-maximum-possible (POMP) scores were used to describe domain prominence, and FDR correction was applied across the 14 domain–biomarker tests (Supplementary Table S7). Because the domains were modeled separately and represent correlated components of the same adapted scale, their coefficients were interpreted as descriptive association patterns rather than mutually independent domain effects.

Bootstrap and permutation procedures used NumPy’s default_rng with a base random seed of 2026 and deterministic analysis-specific offsets to ensure reproducibility; permutation analyses used 20,000 repetitions where required. The statistical analysis code is not deposited publicly with the manuscript but is available from the corresponding author upon reasonable request, subject to applicable institutional and data-governance considerations.

## 3. Results

### 3.1. Study Sample Profile and Variable Distributions

The analytic sample comprised 203 doctoral research scholars, including 123 females (60.6%) and 80 males (39.4%). Mean age was 27.19 years (SD 1.51; range 2433). Most participants were urban (68.5%), and 70.0% were classified as sedentary. After combining the single lower-category observation with the middle category for descriptive reporting, 193 participants (95.1%) were in the lower/middle socioeconomic group and 10 (4.9%) in the upper group. Mean BMI was 22.54 kg/m^2^ (SD 3.02). Complete participants’ characteristics are summarized in Table 1.

Mean AST was 109.52 (SD 21.04; observed range 51–157). Mean DASS-21 depression, anxiety, and stress scores were 13.10 (SD 7.90), 14.68 (SD 7.50), and 15.97 (SD 7.57), respectively, and mean PSQI was 7.73 (SD 2.86). Above-normal DASS-21 scores were observed in 131 participants (64.5%) for depression, 176 (86.7%) for anxiety, and 100 (49.3%) for stress; 161 participants (79.3%) had poor sleep quality (PSQI > 5). The complete five-level symptom-severity distribution is shown in Table 2. Moderate-to-extremely severe ranges were present in 88 participants (43.3%) for depression, 151 (74.4%) for anxiety, and 58 (28.6%) for stress. Extremely severe anxiety symptoms were observed in 54 participants (26.6%). These are questionnaire-defined symptom-severity categories and should not be interpreted as prevalence estimates of diagnosed psychiatric disorders.

**Figure 1:**
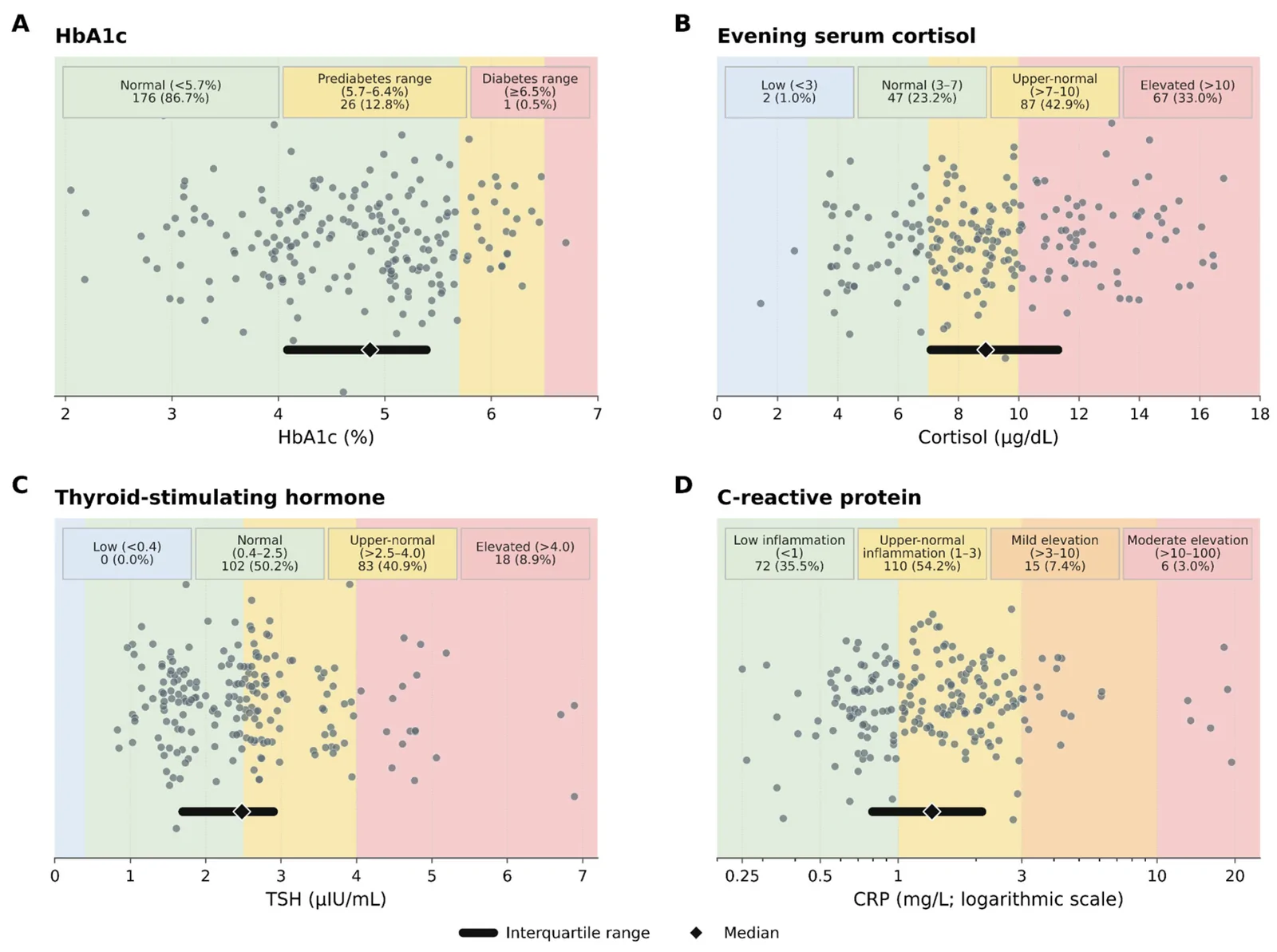
Distribution of biochemical markers in the study sample (*N* = 203) across predefined concentration categories. Individual points represent participant values; diamonds indicate medians and horizontal bars indicate interquartile ranges. HbA1c categories follow established diagnostic thresholds. For evening serum cortisol, TSH, and CRP, the displayed categories represent literature-informed reference or interpretive concentration bands used for descriptive contextualization of the study sample and should not be interpreted as laboratory-specific diagnostic cut-offs or as evidence of endocrine, thyroid, or inflammatory disease. For CRP, the terminology shown reflects progressively higher concentrations associated with inflammatory activity in the cited literature; CRP is a nonspecific acute-phase reactant, and these bands do not represent universally applicable clinical severity grades. No participant had a CRP concentration > 100 mg/L; therefore, the >100 mg/L category defined in the Methods is not displayed in the figure.

Mean HbA1c was 4.71% (SD 0.95), mean evening serum cortisol was 9.08 µg/dL (SD 3.20), and mean TSH was 2.55 µIU/mL (SD 1.10). CRP was right-skewed, with a median of 1.35 mg/L (IQR 0.80–2.11). Descriptively, 176 participants (86.7%) had HbA1c < 5.7%, 26 (12.8%) had values of 5.7–6.4%, and 1 (0.5%) had HbA1c ≥ 6.5%. Using the predefined biomarker-specific descriptive/reference bands, 67 participants (33.0%) had evening cortisol > 10 µg/dL, 18 (8.9%) had TSH > 4.0 µIU/mL, and 21 (10.3%) had CRP > 3 mg/L. These percentages reflect biomarker-specific concentration bands and were not directly compared because the underlying reference or interpretive categories are not clinically equivalent across biomarkers. The complete distributions are presented in Figure 1.

### 3.2. Academic Stress and Biochemical Markers

**Figure 2:**
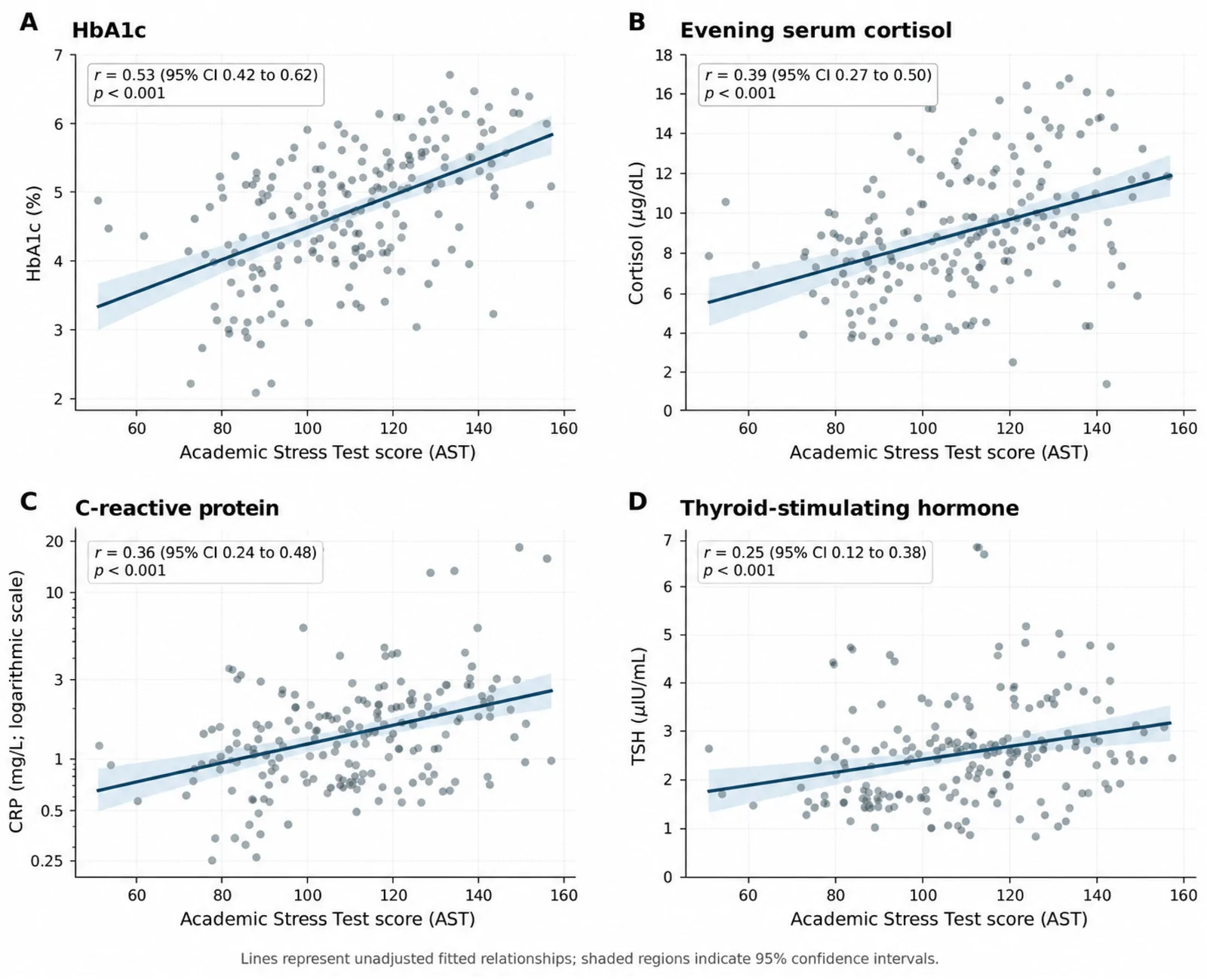
Unadjusted associations of total Academic Stress Scale (AST) score with HbA1c, evening serum cortisol, CRP, and TSH. Points represent participants; lines show fitted unadjusted relationships with 95% confidence bands. CRP is displayed in raw mg/L on a logarithmic axis, while correlation and regression analyses used ln(CRP).

Higher AST was positively associated with all four biochemical markers in unadjusted analyses (Figure 2), with the strongest correlation observed for HbA1c, followed by evening cortisol, CRP, and TSH (all *p* < 0.001). Spearman sensitivity correlations showed the same direction and broadly similar magnitudes across all four biomarkers (all *p* < 0.001).

**Figure 3:**
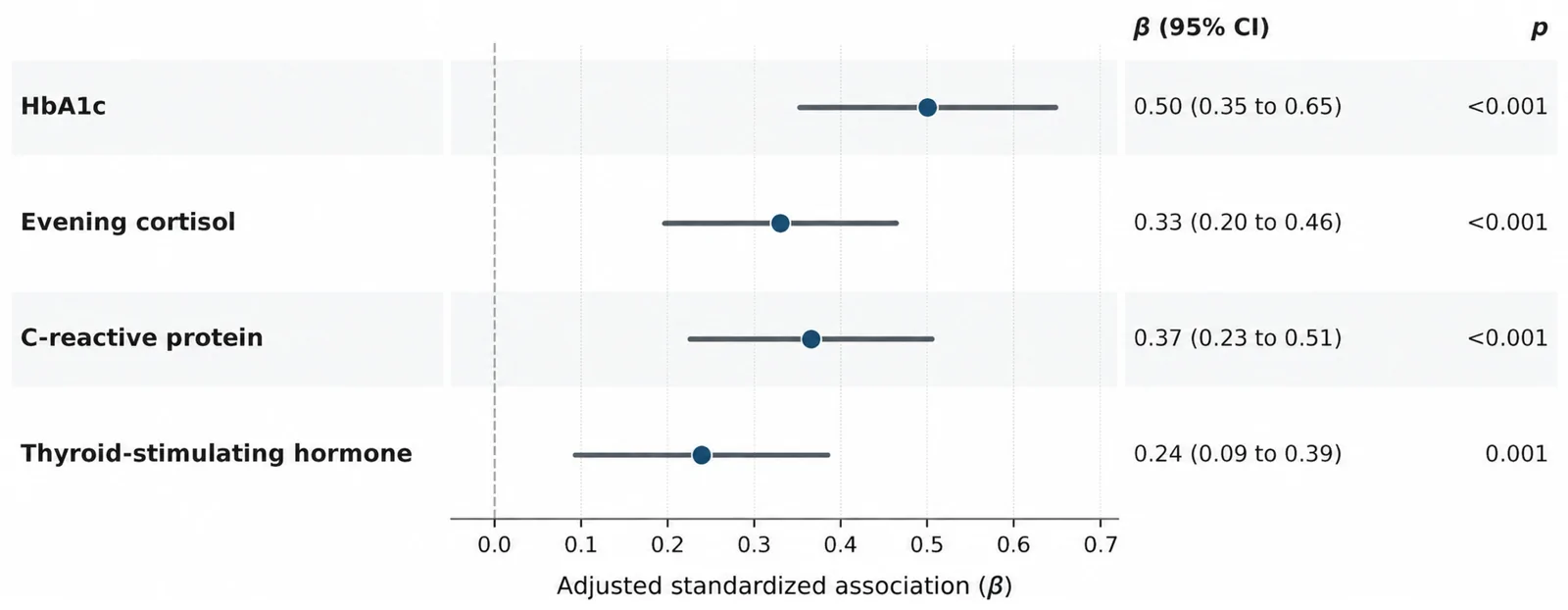
Adjusted standardized associations between academic stress and biochemical markers. Points are standardized AST coefficients from separate HC3-robust linear models adjusted for age, gender, BMI, and lifestyle activity; lines are 95% confidence intervals. CRP was natural-log transformed before modeling.

Associations remained after adjustment for age, gender, BMI, and lifestyle activity (Table 3; Figure 3). A 10-point higher AST score was associated with 0.225 percentage points higher HbA1c (95% CI 0.158–0.291), 0.502 µg/dL higher evening cortisol (0.299–0.706), 13.9% higher CRP (approximately 8.4–19.7%), and 0.125 µIU/mL higher TSH (0.049–0.200); all four associations remained statistically supported after FDR correction (*q* ≤ 0.0013). For clinical context, the adjusted HbA1c difference of 0.225 percentage points represents approximately 28% of the 0.8-percentage-point interval between the conventional 5.7% prediabetes and 6.5% diabetes thresholds. This comparison is intended only to contextualize the magnitude of the association and should not be interpreted as a within-person increase in HbA1c or as evidence of progression between diagnostic categories. Standardized adjusted estimates and complete model results are provided in Table 3 and Supplementary Table S2.

### 3.3. Robustness and Sensitivity Analyses

Diagnostic assessment identified heteroscedasticity for HbA1c (Breusch–Pagan *p* = 0.009) and departures from residual normality for HbA1c, ln(CRP), and TSH (Jarque–Bera *p* ≤ 0.007); HC3-robust inference and outcome-specific sensitivity analyses were therefore used. The RESET test suggested a possible model-specification departure for cortisol (*p* = 0.040), whereas quadratic AST terms were not supported for any biomarker (HbA1c *p* = 0.268, cortisol *p* = 0.692, ln(CRP) *p* = 0.843, TSH *p* = 0.476). Because RESET is an omnibus specification test and does not identify the source of departure, additional checks were performed for the cortisol model. Simultaneously adding quadratic terms for AST, age, and BMI did not provide evidence of nonlinear effects (joint Wald *p* = 0.579). In a sensitivity model using ln(cortisol), the RESET test was no longer statistically significant (*p* = 0.133), while the AST association remained positive; each 10-point higher AST corresponded to approximately 5.2% higher cortisol (95% CI 2.3–8.0%). Variance-inflation factors ranged from 1.06 to 1.13. Several observations exceeded the 4/N Cook’s-distance screening threshold, but the maximum Cook’s distance was 0.103. No observations were deleted on this basis, and influence-resistant robust regression produced closely aligned AST estimates (Table 4).

Robust M-estimation yielded AST coefficients closely aligned with the corresponding HC3-OLS estimates: 0.0224 for HbA1c, 0.0530 for cortisol, 0.0119 for ln(CRP), and 0.00532 for ln(TSH), each *p* < 0.001. Excluding nine participants with HbA1c < 3.0% reduced the HbA1c coefficient from 0.0225 to 0.0170 per AST point (95% CI 0.0110–0.0229) but did not remove the association. Modeling CRP with a Gamma distribution and log link likewise supported a positive association; each 10-point higher AST corresponded to approximately 17.1% higher CRP (95% CI 10.3–24.2%). In the separate HC3-OLS transformation sensitivity analysis, natural-log transformation of TSH also retained the AST association (*B* = 0.00489 per AST point, 95% CI 0.00217–0.00761; *p* < 0.001).

Additional adjustment for urban/rural demography produced minimal changes in the AST coefficients. For HbA1c, adding PSQI attenuated the AST coefficient from 0.0225 to 0.0178 per point (95% CI 0.0112–0.0244), whereas adding eating-window duration produced essentially no change (*B* = 0.0224, 95% CI 0.0157–0.0291). The association remained when both were added simultaneously. For cortisol, additional PSQI adjustment also retained the AST association (*B* = 0.0488, 95% CI 0.0248–0.0728). Because these variables were measured concurrently and may themselves be stress-responsive, these models are interpreted as sensitivity adjustments rather than evidence of confounding or mediation.

### 3.4. Psychological, Sleep, and Gender Patterning

**Figure 4:**
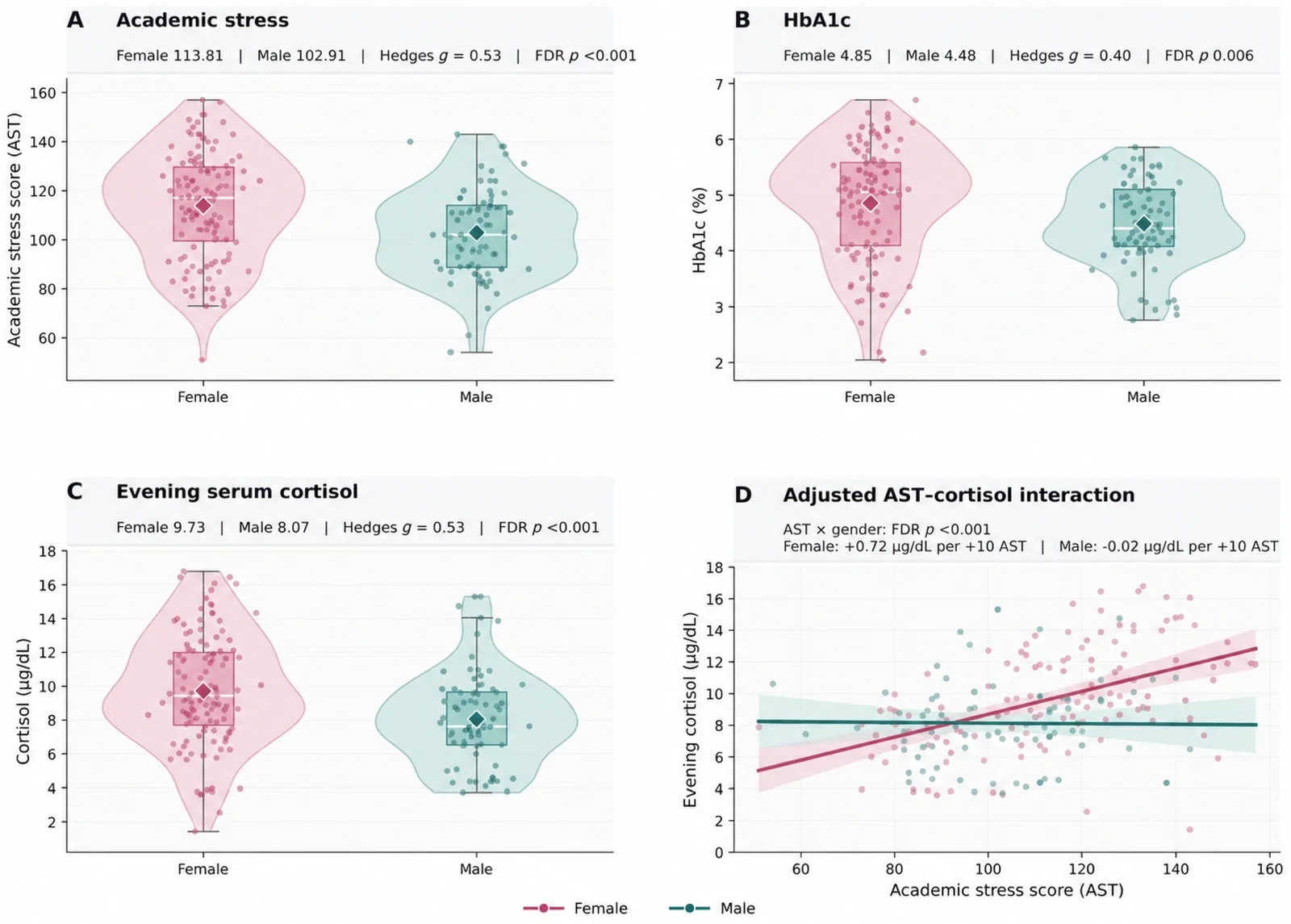
Gender patterning of academic stress and key biochemical markers. Panels (**A**–**C**) show participant distributions of AST, HbA1c, and evening serum cortisol by gender, with mean-based Hedges’ *g* summaries and FDR-adjusted *p* values. Diamonds indicate group means in Panels (**A**–**C**). Panel (**D**) shows the planned exploratory adjusted gender-specific AST–cortisol slopes, with shaded regions indicating 95% confidence intervals. The interaction provides exploratory evidence of gender heterogeneity in the AST–cortisol association but should not be interpreted as evidence of a physiological sex-specific mechanism.

Gender-stratified analyses showed higher mean AST among female than male participants (113.81 vs. 102.91; Hedges’ *g* = 0.53, FDR *q* < 0.001) (Figure 4A). Female participants also had higher mean HbA1c (4.85% vs. 4.48%; *g* = 0.40, *q* = 0.006) and evening cortisol (9.73 vs. 8.07 µg/dL; *g* = 0.53, *q* < 0.001), whereas differences in ln(CRP) and ln(TSH) were not supported (Figure 4B,C). Detailed continuous and binary gender comparisons are provided in Supplementary Table S3A,B.

**Figure 5:**
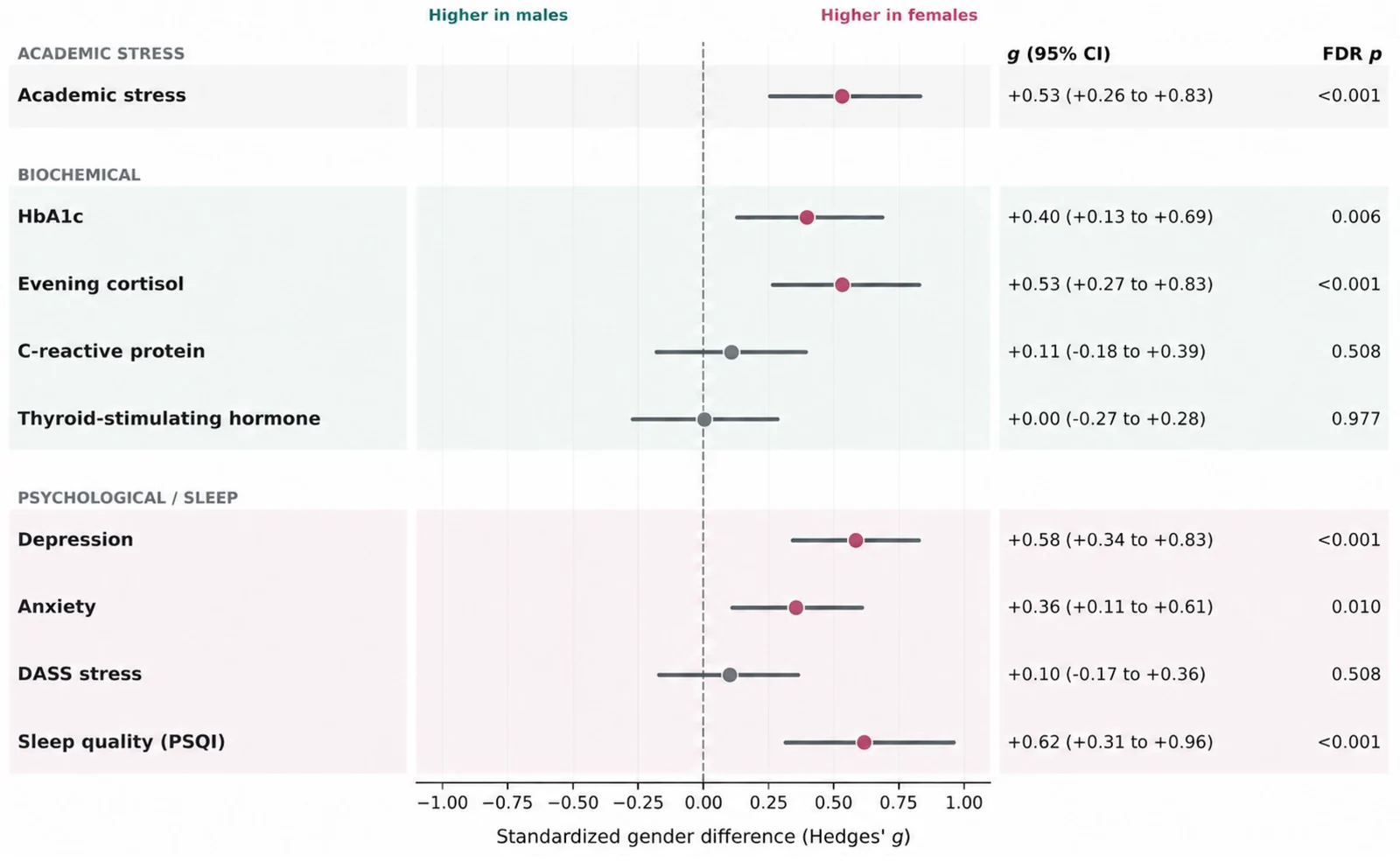
Comprehensive standardized gender profile across academic, biochemical, psychological, and sleep outcomes. Positive Hedges g values indicate higher mean values in females; negative values indicate higher mean values in males. Rose-colored points denote estimates whose bootstrap 95% confidence interval excludes zero in the female-higher direction; grey points denote confidence intervals that include zero. No estimate had a confidence interval entirely below zero. FDR-adjusted *p* values are shown in the right column. Confidence intervals for Hedges *g* were estimated using 5000 bootstrap resamples. CRP and TSH were natural-log transformed for gender comparisons; FDR correction was applied across all nine continuous comparisons.

Among psychological and sleep measures, females had higher mean depression, anxiety, and PSQI scores, whereas the difference in DASS-21 stress symptoms was not supported. Poor sleep was also more frequent among females than males (85.4% vs. 70.0%; prevalence ratio 1.22, 95% CI 1.04–1.43; FDR *q* = 0.022). Detailed binary comparisons and sensitivity analyses are provided in Figure 5 and Supplementary Table S3A,B.

Five-level DASS-21 severity distributions showed gender differences for depression (*χ^2^*(4) = 20.50, FDR q = 0.0012; Cramer’s *V* = 0.318) and anxiety (*χ^2^*(4) = 15.33, *q* = 0.0061; *V* = 0.275), but not stress (*χ^2^*(4) = 2.90, *q* = 0.575; *V* = 0.119). Females were more represented in the higher-severity categories for depression and anxiety, including the extremely severe categories. The complete severity distributions are provided in Supplementary Table S4.

### 3.5. Exploratory Psychological Symptom-Biochemical Associations

Supplementary Spearman analyses showed consistent positive associations between DASS-21 symptom scores and all four biochemical markers, with all 12 subscale–biomarker correlations remaining statistically supported after FDR correction (all *q* < 0.002; Supplementary Table S5). The largest correlations were observed for HbA1c (*ρ* = 0.535–0.579), while correlations with evening cortisol, CRP, and TSH ranged from *ρ* = 0.386–0.489, 0.274–0.307, and 0.221–0.252, respectively. A robustness analysis using the DASS-21 total symptom score showed the same overall pattern (*ρ* = 0.270–0.668; all *q* < 0.001). These unadjusted findings characterize broader psychological symptom burden and do not establish independent or causal psychological–biochemical relationships.

### 3.6. Exploratory Gender Modification of AST–Biomarker Associations

In the planned interaction analyses, statistically supported evidence of gender heterogeneity was observed for the AST–cortisol association (AST-by-male interaction *B* = −0.0744 µg/dL per AST point, 95% CI −0.1131 to −0.0356; FDR *q* < 0.001; Figure 4D; Supplementary Table S6). The adjusted AST–cortisol association was positive in females and essentially null in males, and the interaction remained stable in robust-regression sensitivity analysis. No supported interaction was observed for HbA1c or ln(CRP), while the ln(TSH) interaction was not retained under robust regression and was therefore considered unstable. Accordingly, the AST–cortisol result is interpreted as statistically supported evidence of gender heterogeneity in the observed association, without implying a physiological sex-specific mechanism.

### 3.7. Exploratory Academic-Stress Domain Profile

**Figure 6:**
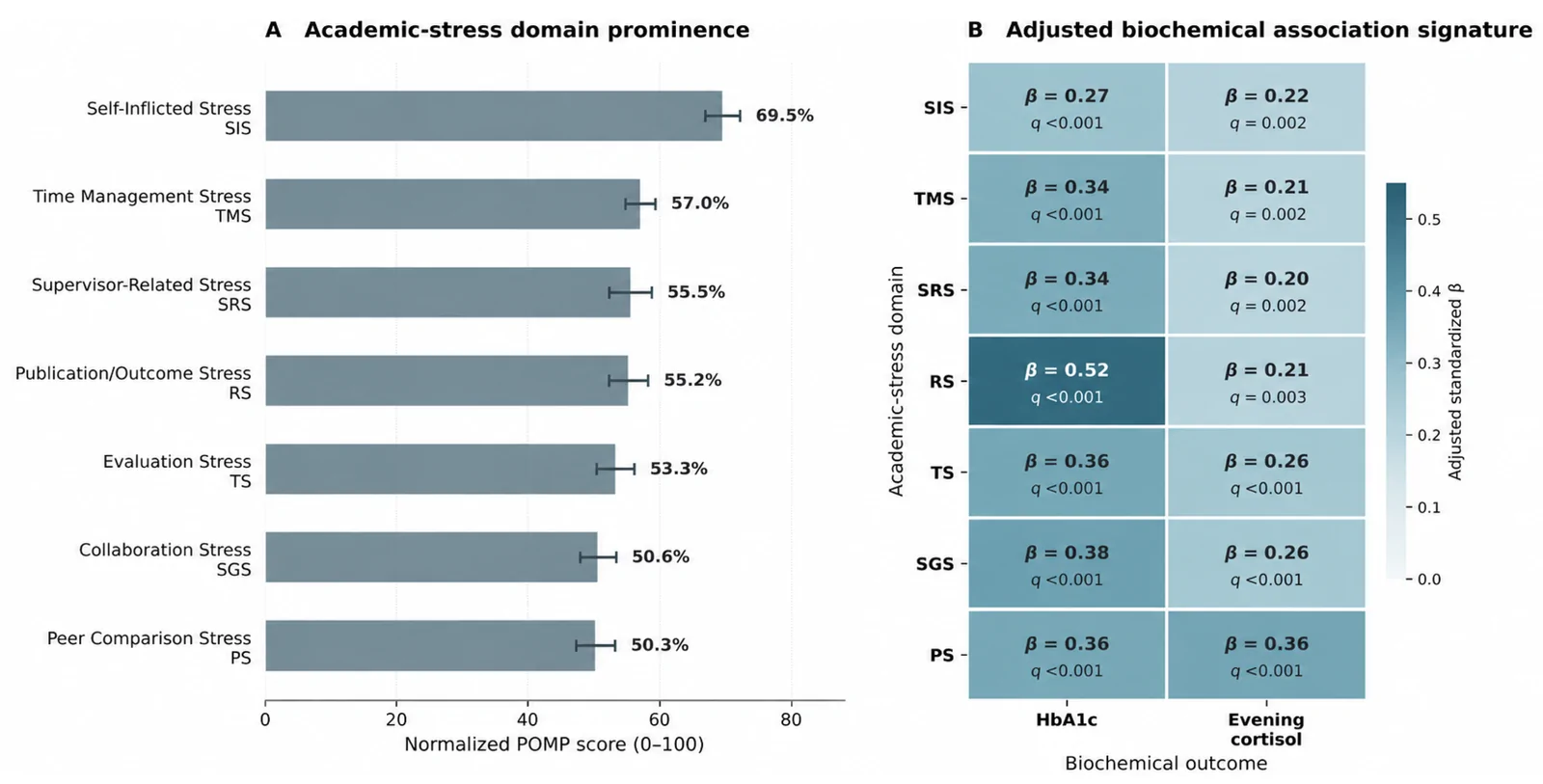
Academic-stress domain prominence and biochemical association signature. Panel (**A**) shows percentage-of-maximum-possible (POMP) domain scores with 95% confidence intervals; higher values indicate greater normalized domain prominence. Panel (**B**) shows standardized coefficients from separate HC3-robust models for HbA1c and evening cortisol adjusted for age, gender, BMI, and lifestyle activity. *q* denotes Benjamini–Hochberg FDR-adjusted *p* value across all 14 domain–biomarker tests. Because domains were modeled separately and represent correlated components of the same scale, differences in coefficient magnitude are presented descriptively and should not be interpreted as evidence that one domain has an independently stronger association with the biomarker outcome.

Normalized domain scores and domain–biomarker associations are summarized in Figure 6 and Supplementary Table S7. Self-Inflicted Stress had the highest mean POMP score (69.5%, 95% CI 66.9–72.1%). All 14 domain–biomarker associations remained statistically supported after FDR correction when domains were modeled separately. Among these models, Publication/Outcome Stress had the largest standardized coefficient for HbA1c (*β* = 0.520), whereas Peer Comparison Stress had the largest standardized coefficient for evening cortisol (*β* = 0.357). Because the domains are correlated components of the same adapted scale, were not mutually adjusted, and the adapted factor structure was not formally evaluated in the present sample, these coefficient differences are descriptive and the domain-level findings should be regarded as preliminary.

## 4. Discussion

In this cross-sectional study of 203 doctoral research scholars, higher academic stress was associated with higher HbA1c, evening serum cortisol, CRP, and TSH after adjustment for age, gender, BMI, and lifestyle activity. The largest standardized association was observed for HbA1c, followed by CRP and cortisol, with a smaller association for TSH. The direction and approximate magnitude of these associations were broadly consistent across rank-based analyses, HC3-robust inference, influence-resistant regression, alternative CRP and TSH specifications, and selected sensitivity adjustments. The study sample also showed substantial psychological symptom burden and poor sleep, while gender-related, DASS-biomarker, and academic-stress domain analyses were treated as secondary or exploratory findings and are interpreted accordingly below.

The HbA1c association warrants particular consideration because HbA1c integrates glycaemic exposure across preceding weeks to months and is less sensitive than a single glucose measurement to immediate food intake or momentary stress. A 10-point difference in AST was associated with approximately 0.225 percentage points higher HbA1c after core adjustment. In the present study, AST ratings referred to participants’ experiences during doctoral training rather than to a fixed short-term recall interval. Accordingly, the broader temporal reference frame of the AST does not correspond precisely to the preceding weeks to months represented by HbA1c, which limits inference about the temporal alignment between academic-stress exposure and glycaemic change. This estimate was attenuated but remained present after excluding very low HbA1c values and after additional adjustment for PSQI. The attenuation with PSQI is compatible with shared variance among academic stress, sleep, and glycaemic regulation. Sleep disturbance could precede, follow, or co-develop with perceived academic stress, and both may share unmeasured determinants. Accordingly, the attenuation after PSQI adjustment should not be interpreted as evidence of mediation or confounding because these variables were measured concurrently.

The magnitude of the AST-HbA1c association should also be interpreted against an inconsistent wider literature. In a recent prospective study sample of healthy young Danish adults, perceived stress and several psychosocial stressors showed only small and statistically inconclusive associations with HbA1c measured approximately one year later [10]. A 2026 systematic review and meta-analysis similarly found little consistent evidence that perceived stress is associated with most conventional metabolic parameters, although insulin resistance showed an exploratory positive association [11]. Rather than indicating a universal stress-metabolic relationship, these findings suggest that associations may vary according to the type and context of stress exposure, population, and study design. A doctoral-specific instrument may capture recurring research pressures not represented by global perceived-stress scales. Conversely, the cross-sectional nature of our study may inflate apparent coherence between concurrently measured constructs and cannot determine whether higher stress preceded higher HbA1c. Prospective studies with repeated assessments of both academic stress and HbA1c are therefore needed to establish whether higher academic stress precedes subsequent differences in glycaemic exposure.

The cortisol association is biologically plausible but should be interpreted within the constraints of cortisol measurement. The HPA axis is dynamic, circadian, and responsive to acute as well as chronic inputs [8,33]. Blood collection was targeted to around 18:00 to reduce variation in sampling time, yet a single evening serum concentration cannot estimate diurnal slope, awakening response, cumulative secretion, or chronic HPA activity. The positive adjusted association with AST therefore indicates that participants reporting greater academic stress tended to have higher cortisol at the evening sampling point; it does not demonstrate persistent hypercortisolism.

The gender-stratified cortisol finding warrants specific consideration. Female participants had higher mean AST and cortisol, and the adjusted AST-cortisol slope was positive in females but essentially null in males. This interaction remained stable with robust regression, unlike the TSH interaction, which did not. However, the finding should be interpreted cautiously because several potentially relevant factors were not systematically measured, including smoking, caffeine intake, alcohol use, medication use, recent exercise, menstrual cycle phase, reproductive hormones, contraceptive use, and other medical or physiological conditions. The finding should therefore be described as gender-stratified heterogeneity rather than evidence of a mechanistic sex difference and is best viewed as a replication target for studies designed specifically around gender- and sex-related modifiers of HPA function.

CRP showed a moderate standardized association with academic stress. Natural-log transformation, HC3 inference, and a Gamma-log model yielded broadly concordant results, indicating that the direction of association was not dependent on a single statistical specification. CRP is nevertheless nonspecific and can vary with infection, adiposity, recent exertion, oral health, and other physiological processes. The present data therefore support an association with systemic inflammatory variation but do not justify claims of stress-induced inflammation or inflammatory disease. This interpretation is consistent with the broader literature emphasizing substantial heterogeneity in physiological biomarkers of chronic stress [9]. Future studies could strengthen inference through repeated sampling and a broader inflammatory panel, including high-sensitivity CRP where low-grade inflammatory variation is the specific target.

The TSH association was smaller than those for HbA1c, CRP, and cortisol but remained statistically supported under both raw and log-transformed specifications. Experimental research has shown that acute psychosocial stress can transiently alter TSH in healthy participants [12], and HPA-HPT interactions are biologically plausible. Yet TSH is influenced by circadian timing, iodine status, thyroid autoimmunity, illness, medications, and individual set points. The current result should therefore be regarded as a modest association with thyroid-axis variation, not evidence that academic stress causes thyroid dysfunction. This distinction is important because most participants were within or near conventional reference ranges and the study did not include free T4, free T3, or thyroid-antibody measures.

The psychological and sleep findings place the biochemical results in a behavioral-health context. Nearly four-fifths of the study sample had PSQI scores above the conventional poor-sleep threshold, while 64.5%, 86.7%, and 49.3% had above-normal depression, anxiety, and stress scores, respectively. These were screening-scale categories rather than clinical diagnoses. The pattern is consistent with the broader doctoral mental-health literature, including the meta-analysis by Chi et al. (2023) [2], doctoral-specific observational studies [3,4], and longitudinal administrative data [5]. The present study adds biochemical characterization within the same study sample rather than relying exclusively on questionnaire outcomes.

Gender-specific DASS findings were secondary to the primary biomarker analyses. Female participants had higher mean depression and anxiety scores, while stress severity did not differ significantly by gender. This pattern is broadly consistent with recent doctoral-student research reporting higher anxiety and depression symptoms among female doctoral students [34]. The five-level distributions provided additional descriptive information on symptom-severity composition, but these findings are not interpreted as evidence of differential psychiatric risk.

The exploratory DASS-biomarker correlations similarly provide contextual rather than mechanistic information. Each DASS domain was positively correlated with each biomarker after FDR correction, with the largest coefficients consistently involving HbA1c and followed by cortisol. Because these analyses were unadjusted and the measures were assessed concurrently, they cannot establish associations independent of academic stress or a causal sequence from academic stress to psychological symptoms and biomarkers.

The academic-stress domain analysis was also exploratory. Self-Inflicted Stress had the highest normalized mean domain score, whereas Publication/Outcome Stress showed the largest standardized association with HbA1c and Peer Comparison Stress the largest association with cortisol. Because the domains are correlated components of an adapted instrument and were modeled separately, these findings should be regarded as exploratory rather than evidence of independent domain-specific effects.

Several features strengthen the study. First, the analysis was focused on four biochemical outcomes, providing a clear exposure-outcome framework while retaining psychological and sleep measures as contextual variables. Second, biochemical measurements were obtained using established automated laboratory platforms, and blood collection was targeted to a consistent evening sampling time. Third, the statistical analysis emphasized effect estimates and confidence intervals, HC3-robust inference, multiplicity control, diagnostics, alternative outcome specifications, and influence-resistant sensitivity models rather than reliance on a single *p* value. The secondary and exploratory analyses were also explicitly identified as such, with FDR correction applied to the relevant test families. Fourth, the adapted academic-stress instrument showed high total internal consistency and acceptable-to-high domain coefficients in the available reliability dataset, without treating these estimates as full psychometric validation.

Several limitations should be considered. The cross-sectional design prevents determination of temporal direction and is compatible with reverse or bidirectional relationships. The single-center, volunteer sample limits generalizability, and the sample was young and relatively homogeneous socioeconomically. Although the adapted academic-stress instrument showed high internal consistency, its construct validity and factor structure were not formally re-evaluated in the present sample. Therefore, the domain-level biomarker findings should be regarded as preliminary evidence that warrants confirmation in independent studies. Academic stress, psychological symptoms, sleep, dietary timing, and lifestyle activity were self-reported. Physical activity was assessed using a study-specific, relatively coarse classification rather than a validated physical-activity instrument. Although exercise frequency and habitual daily inactivity were captured, exercise duration and intensity were not separately quantified; therefore, residual confounding related to physical-activity dose cannot be excluded, particularly for metabolic and inflammatory biomarkers. Detailed medication use beyond the prespecified eligibility exclusions, smoking status, and acute infection status were not systematically characterized. A single evening cortisol measurement cannot characterize diurnal HPA function. Menstrual phase, hormonal contraception, sleep–wake timing, recent exercise, caffeine intake, and other short-term influences on endocrine measures were not systematically recorded. HbA1c may be affected by hematologic and red-cell conditions not fully characterized here, although the association remained after exclusion of very low values.

These considerations define the appropriate interpretation of the findings. The data do not support routine biochemical screening of doctoral students and do not establish that academic stress causes metabolic, endocrine, inflammatory, or thyroid disease. From an institutional perspective, the wider literature supports attention to modifiable academic conditions such as workload, supervisory clarity, publication expectations, uncertainty, social comparison, sleep, and access to mental-health support [6,7,35,36]. The next research step could be longitudinal, multisite assessment beginning early in doctoral training, with repeated measures of academic stress, psychological symptoms, sleep, activity, HbA1c, diurnal or longer-term cortisol indices, inflammatory markers, and thyroid function. Such designs would help distinguish stable between-person differences from within-person change and provide the temporal structure required for credible pathway analysis.

## 5. Conclusion

Higher academic stress in doctoral research scholars was associated with higher HbA1c, evening serum cortisol, CRP, and TSH after adjustment for age, gender, BMI, and lifestyle activity, with broadly consistent findings across sensitivity analyses. The accompanying psychological symptom burden and poor sleep provide relevant behavioral-health context, but the secondary and interaction analyses should not be interpreted as evidence of specificity, independence, mediation, or causal direction. The findings indicate an association between academic stress and variation across glycaemic, neuroendocrine, inflammatory, and thyroid-related markers, while the cross-sectional design limits conclusions regarding temporal direction or causality. Longitudinal multisite studies with repeated psychological, sleep, and biochemical measurements are needed to clarify temporal relationships and determine whether the observed patterns persist or change over doctoral training.

## Figures and Tables

**Table 1:** Characteristics of the analytic sample (*N* = 203). Values are mean ± SD, *n* (%), or median [IQR] as indicated.

Characteristic	Summary
*N*	203
Age, years	27.19 ± 1.51
Female/Male	123 (60.6%)/80 (39.4%)
Urban/Rural	139 (68.5%)/64 (31.5%)
Sedentary/Active lifestyle	142 (70.0%)/61 (30.0%)
Socioeconomic category	Lower/middle 193 (95.1%); Upper 10 (4.9%)
BMI, kg/m^2^	22.54 ± 3.02
Self-reported eating window (hours)	12.09 ± 2.06
Academic Stress Scale (AST)	109.52 ± 21.04; median 111; range 51–157
DASS-21 depression	13.10 ± 7.90
DASS-21 anxiety	14.68 ± 7.50
DASS-21 stress	15.97 ± 7.57
PSQI	7.73 ± 2.86; poor sleep 161 (79.3%)
HbA1c, %	4.71 ± 0.95
Evening serum cortisol, µg/dL	9.08 ± 3.20
TSH, µIU/mL	2.55 ± 1.10
CRP, mg/L	1.35 [0.80–2.11], median [IQR]

**Table 2:** DASS-21 symptom-severity distribution in the total sample (*N* = 203). Severity categories were derived from the conventional doubled DASS-21 subscale scores and represent screening-scale symptom ranges rather than psychiatric diagnoses.

DASS-21 Severity	Depression, *n* (%)	Anxiety, *n* (%)	Stress, *n* (%)
Normal	72 (35.5%)	27 (13.3%)	103 (50.7%)
Mild	43 (21.2%)	25 (12.3%)	42 (20.7%)
Moderate	54 (26.6%)	65 (32.0%)	29 (14.3%)
Severe	21 (10.3%)	32 (15.8%)	25 (12.3%)
Extremely severe	13 (6.4%)	54 (26.6%)	4 (2.0%)

**Table 3:** Unadjusted and adjusted associations between academic stress and biochemical markers. Adjusted models include age, gender, BMI, and lifestyle activity and use HC3-robust 95% confidence intervals. FDR *q* values reflect Benjamini–Hochberg correction across the four biomarker models.

Outcome	Unadjusted *r* (95% CI)	Adjusted Effect per +10 AST (95% CI)	Standardized *β* (95% CI)	FDR *q*	Adjusted *R*^2^
HbA1c	0.528 (0.421–0.621)	+0.225 percentage points (0.158–0.291)	0.501 (0.353–0.649)	<0.001	0.270
Evening cortisol	0.392 (0.269–0.502)	+0.502 µg/dL (0.299–0.706)	0.330 (0.197–0.464)	<0.001	0.183
CRP, ln-transformed	0.362 (0.236–0.476)	+13.9% (8.4–19.7%)	0.366 (0.226–0.506)	<0.001	0.149
TSH	0.253 (0.120–0.378)	+0.125 µIU/mL (0.049–0.200)	0.240 (0.094–0.385)	0.0013	0.111

**Table 4:** Selected robustness and sensitivity analyses for the AST-biomarker associations. These analyses were used to assess stability and were not treated as alternative primary models.

Sensitivity Analysis	Estimate for AST	95% CI/Interpretation	Result
HbA1c: robust M-estimation	*B* = 0.02239 per AST point	SE = 0.00298	*p* < 0.001
HbA1c: exclude values < 3.0% (*N* = 194)	*B* = 0.01699 per point	0.01103–0.02294	*p* < 0.001
HbA1c: +PSQI	*B* = 0.01782 per point	0.01122–0.02442	*p* < 0.001
HbA1c: +eating-window duration	*B* = 0.02243 per point	0.01573–0.02913	*p* < 0.001
Cortisol: robust M-estimation	*B* = 0.05301 per point	SE = 0.01069	*p* < 0.001
Cortisol: +PSQI	*B* = 0.04879 per point	0.02478–0.07280	*p* < 0.001
CRP: robust M-estimation, ln outcome	*B* = 0.01191 per point	SE = 0.00223	*p* < 0.001
CRP: Gamma model, log link	*B* = 0.01576 per point	~17.1% higher CRP per +10 AST (10.3–24.2%)	*p* < 0.001
TSH: robust M-estimation, ln(TSH) outcome	*B* = 0.00532 per point	SE = 0.00135	*p* < 0.001
TSH: ln-transformed outcome, HC3-OLS	*B* = 0.00489 per point	0.00217–0.00761	*p* < 0.001
Quadratic AST terms	HbA1c *p* = 0.268; cortisol *p* = 0.692; CRP *p* = 0.843; TSH *p* = 0.476	No supported departure from linearity	All non-significant

## Data Availability

The datasets obtained in this study are not publicly accessible due to the presence of identifying human participant information and are governed by institutional ethical and privacy regulations. De-identified data that corroborate the findings of this study can be obtained from the corresponding author upon reasonable request, contingent upon permission from the Institutional Ethics Committee and relevant institutional rules.

## Supplementary Materials

The following supporting information can be downloaded at: https://www.mdpi.com/article/10.3390/healthcare14193280/s1, Table S1: Structure and internal consistency of the doctoral-adapted Academic Stress Scale in the available item-level reliability dataset (*n* = 39). Coefficients are reported as reliability estimates and not as factor-analytic validation; Table S2: Full covariate coefficients from the four adjusted biochemical models. Female and sedentary are reference categories. All models use HC3-robust standard errors. Intercepts are omitted from this display for compactness; Table S3A: Continuous gender comparisons. Hedges’ *g* is positive when the female mean is higher. Confidence intervals were estimated by 5000 bootstrap resamples; *q* values are FDR adjusted across nine comparisons; Table S3B: Gender-stratified prevalence comparisons for descriptive threshold-based outcomes. Thresholds are screening/reference categories and not clinical diagnoses; Table S4: DASS-21 five-level symptom-severity distribution in the total cohort and by gender. Overall, 2 × 5 gender-distribution tests are reported once per domain; *q* values are Benjamini–Hochberg adjusted across the three DASS domains and *V* denotes Cramer’s *V*. Severity categories are screening ranges, not psychiatric diagnoses; Table S5: DASS-21 symptom scores and biochemical markers: Spearman associations between DASS-21 symptom scores and biochemical markers. Panel A presents the prespecified subscale analyses, and Panel B presents the supplementary robustness analysis using the DASS-21 total symptom score. Confidence intervals were estimated using 5000 bootstrap resamples. Benjamini–Hochberg FDR adjustment was applied separately across the 12 subscale–biomarker comparisons in Panel A and the four total-score–biomarker comparisons in Panel B. These analyses are unadjusted and do not establish independent or causal psychological–biochemical relationships. The DASS-21 total score was used only as a composite measure of overall symptom burden and was not interpreted as an independent diagnostic construct; Table S6: Adjusted AST-by-gender interactions for biochemical outcomes. Models include centered AST, gender, their interaction, age, BMI, and lifestyle activity. Female is the reference group. Slopes are expressed per one AST point; Table S7: Exploratory academic-stress domain prominence and adjusted standardized associations with HbA1c and cortisol. Each domain–biomarker combination was modeled separately with age, gender, BMI, and lifestyle activity; *q* values are FDR adjusted across all 14 tests.
