# Supplementary material for: Academic Stress in Doctoral Research Scholars in Relation to Biochemical Markers and Mental Health Symptoms: A Single-Center Cross-Sectional Study
Source: Healthcare (Basel). 2026 Oct 2;14(19):3280. doi: 10.3390/healthcare14193280 (PMC13665388; doi:10.3390/healthcare14193280)
Supplement: Supplementary file 1 [file healthcare-14-03280-s001.zip › healthcare-4579969-supplementary.pdf]

Supplementary Material

Supplementary Table S1. Structure and internal consistency of the doctoral-adapted Academic Stress Scale in the available item-level reliability dataset (n=39). Coefficients are reported as reliability estimates and not as factor-analytic validation.

**Domain**	**Abbreviation**	**Items**	**Theoretical score range**	**Cronbach α (reliability sample n=39)**
Supervisor-Related Stress	SRS	5	5-25	0.885
Publication/Outcome Stress	RS	5	5-25	0.890
Evaluation Stress	TS	5	5-25	0.866
Collaboration Stress	SGS	5	5-25	0.774
Peer Comparison Stress	PS	5	5-25	0.859
Time Management Stress	TMS	5	5-25	0.749
Self-Inflicted Stress	SIS	4	4-20	0.783
Total Academic Stress Scale	AST	34	34-170	0.930

Supplementary Table S2. Full covariate coefficients from the four adjusted biochemical models. Female and sedentary are reference categories. All models use HC3-robust standard errors. Intercepts are omitted from this display for compactness.

**Outcome**	**Predictor**	**B**	**Robust SE**	**95% CI**	**p**
HbA1c	AST	0.02249	0.00339	0.01584 to 0.02913	<0.001
HbA1c	Age	-0.02043	0.03892	-0.09671 to 0.05585	0.600
HbA1c	Male	-0.13678	0.12191	-0.37572 to 0.10217	0.262
HbA1c	BMI	0.02283	0.02168	-0.01967 to 0.06532	0.292
HbA1c	Active	-0.02121	0.13494	-0.28568 to 0.24327	0.875

Evening cortisol	AST	0.05024	0.01038	0.02989 to 0.07059	<0.001
Evening cortisol	Age	-0.23561	0.14084	-0.51164 to 0.04043	0.094
Evening cortisol	Male	-1.01191	0.40427	-1.80426 to -0.21955	0.012
Evening cortisol	BMI	-0.06155	0.06788	-0.19459 to 0.07148	0.365
Evening cortisol	Active	-0.57592	0.46527	-1.48783 to 0.33599	0.216
ln(CRP)	AST	0.01298	0.00253	0.00802 to 0.01794	<0.001
ln(CRP)	Age	0.05909	0.03278	-0.00517 to 0.12334	0.071
ln(CRP)	Male	0.10176	0.10146	-0.09709 to 0.30061	0.316
ln(CRP)	BMI	-0.03448	0.01608	-0.06600 to -0.00297	0.032
ln(CRP)	Active	-0.22224	0.10075	-0.41971 to -0.02477	0.027
TSH	AST	0.01246	0.00387	0.00488 to 0.02005	0.0013
TSH	Age	-0.11568	0.03953	-0.19316 to -0.03820	0.003
TSH	Male	0.20805	0.17123	-0.12756 to 0.54366	0.224
TSH	BMI	-0.04019	0.02041	-0.08019 to -0.00018	0.049
TSH	Active	-0.36159	0.18007	-0.71452 to -0.00865	0.045

Supplementary Table S3A. Continuous gender comparisons. Hedges g is positive when the female mean is higher. Confidence intervals were estimated by 5,000 bootstrap resamples; q values are FDR adjusted across nine comparisons.

**Outcome**	**Female mean**	**Male mean**	**Hedges g (95% CI)**	**FDR q**
Academic stress	113.81	102.91	0.53 (0.26 to 0.83)	<0.001
HbA1c	4.85	4.48	0.40 (0.13 to 0.69)	0.006
Evening cortisol	9.73	8.07	0.53 (0.27 to 0.83)	<0.001
ln(CRP)	0.361	0.279	0.11 (-0.18 to 0.39)	0.508

ln(TSH)	0.851	0.850	0.00 (-0.27 to 0.28)	0.977
Depression	14.86	10.40	0.58 (0.34 to 0.83)	<0.001
Anxiety	15.72	13.08	0.36 (0.11 to 0.61)	0.010
DASS stress	16.28	15.50	0.10 (-0.17 to 0.36)	0.508
PSQI	8.40	6.70	0.62 (0.31 to 0.96)	<0.001

Supplementary Table S3B. Gender-stratified prevalence comparisons for descriptive threshold-based outcomes. Thresholds are screening/reference categories and not clinical diagnoses.

**Categorical outcome**	**Female n/N (%)**	**Male n/N (%)**	**Prevalence ratio (95% CI)**	**FDR q**
HbA1c >=5.7%	26/123 (21.1%)	1/80 (1.3%)	16.91 (2.34-122.16)	<0.001
Cortisol >10 μg/dL	51/123 (41.5%)	16/80 (20.0%)	2.07 (1.28-3.37)	0.006
TSH >4.0 μIU/mL	11/123 (8.9%)	7/80 (8.8%)	1.02 (0.41-2.53)	0.962
CRP >3 mg/L	13/123 (10.6%)	8/80 (10.0%)	1.06 (0.46-2.43)	0.962
Depression above normal	84/123 (68.3%)	47/80 (58.8%)	1.16 (0.93-1.45)	0.330
Anxiety above normal	104/123 (84.6%)	72/80 (90.0%)	0.94 (0.85-1.04)	0.423
DASS stress above normal	63/123 (51.2%)	37/80 (46.3%)	1.11 (0.83-1.48)	0.652
Poor sleep quality	105/123 (85.4%)	56/80 (70.0%)	1.22 (1.04-1.43)	0.022

Supplementary Table S4. DASS-21 five-level symptom-severity distribution in the total cohort and by gender. Overall 2×5 gender-distribution tests are reported once per domain; q values are Benjamini-Hochberg adjusted across the three DASS domains and V denotes Cramer's V. Severity categories are screening ranges, not psychiatric diagnoses.

Domain	Severity	Total n (%)	Female n (%)	Male n (%)	Overall gender test
Depression	Normal	72 (35.5%)	39 (31.7%)	33 (41.2%)	$\chi^2(4)=20.50$; q=0.0012; V=0.318
	Mild	43 (21.2%)	18 (14.6%)	25 (31.2%)	
	Moderate	54 (26.6%)	36 (29.3%)	18 (22.5%)	
	Severe	21 (10.3%)	17 (13.8%)	4 (5.0%)	
	Extremely severe	13 (6.4%)	13 (10.6%)	0 (0.0%)	
Anxiety	Normal	27 (13.3%)	19 (15.4%)	8 (10.0%)	$\chi^2(4)=15.33$; q=0.0061; V=0.275
	Mild	25 (12.3%)	12 (9.8%)	13 (16.2%)	
	Moderate	65 (32.0%)	34 (27.6%)	31 (38.8%)	
	Severe	32 (15.8%)	15 (12.2%)	17 (21.2%)	
	Extremely severe	54 (26.6%)	43 (35.0%)	11 (13.8%)	
Stress	Normal	103 (50.7%)	60 (48.8%)	43 (53.8%)	$\chi^2(4)=2.90$; q=0.575; V=0.119
	Mild	42 (20.7%)	26 (21.1%)	16 (20.0%)	
	Moderate	29 (14.3%)	18 (14.6%)	11 (13.8%)	
	Severe	25 (12.3%)	15 (12.2%)	10 (12.5%)	
	Extremely severe	4 (2.0%)	4 (3.3%)	0 (0.0%)	

Supplementary Table S5. DASS-21 symptom scores and biochemical markers: Spearman associations between DASS-21 symptom scores and biochemical markers. Panel A presents the prespecified subscale analyses, and Panel B presents the supplementary robustness analysis using the DASS-21 total symptom score. Confidence intervals were estimated using 5,000 bootstrap resamples. Benjamini–Hochberg FDR adjustment was applied separately across the 12 subscale–biomarker

comparisons in Panel A and the four total-score–biomarker comparisons in Panel B. These analyses are unadjusted and do not establish independent or causal psychological–biochemical relationships. The DASS-21 total score was used only as a composite measure of overall symptom burden and was not interpreted as an independent diagnostic construct.

Panel A. DASS-21 subscale–biomarker associations

DASS-21 subscale	Biomarker	N	Spearman ρ (95% CI)	FDR q
Depression	HbA1c	203	0.535 (0.425–0.633)	<0.001
Depression	Evening cortisol	203	0.489 (0.368–0.597)	<0.001
Depression	CRP	203	0.300 (0.166–0.426)	<0.001
Depression	TSH	203	0.252 (0.116–0.387)	<0.001
Anxiety	HbA1c	203	0.579 (0.472–0.671)	<0.001
Anxiety	Evening cortisol	203	0.387 (0.252–0.506)	<0.001
Anxiety	CRP	203	0.307 (0.162–0.440)	<0.001
Anxiety	TSH	203	0.221 (0.081–0.354)	0.0016
DASS stress	HbA1c	203	0.540 (0.431–0.641)	<0.001
DASS stress	Evening cortisol	203	0.386 (0.251–0.508)	<0.001
DASS stress	CRP	203	0.274 (0.139–0.406)	<0.001
DASS stress	TSH	203	0.243 (0.105–0.381)	<0.001

Panel B. Supplementary robustness analysis using the DASS-21 total symptom score

DASS-21 measure	Biomarker	N	Spearman ρ (95% CI)	FDR q
Total symptom score	HbA1c	203	0.668 (0.572–0.750)	<0.001
Total symptom score	Evening cortisol	203	0.512 (0.392–0.619)	<0.001
Total symptom score	CRP	203	0.338 (0.196–0.464)	<0.001
Total symptom score	TSH	203	0.270 (0.130–0.408)	<0.001

Supplementary Table S6. Adjusted AST-by-gender interactions for biochemical outcomes. Models include centered AST, gender, their interaction, age, BMI, and lifestyle activity. Female is the reference group. Slopes are expressed per one AST point.

Outcome	AST×male B	95% CI	FDR q	Female AST slope	Male AST slope	Robustness interpretation
HbA1c	-0.00791	-0.02022 to 0.00439	0.277	0.02485	0.01694	No interaction
Evening cortisol	-0.07437	-0.11312 to -0.03562	<0.001	0.07248	-0.00189	Stable in robust regression
ln(CRP)	-0.00388	-0.01378 to 0.00602	0.442	0.01414	0.01026	No interaction
ln(TSH)	0.00663	0.00142 to 0.01184	0.025	0.00291	0.00954	Not stable in robust regression (p=0.074)

Supplementary Table S7. Exploratory academic-stress domain prominence and adjusted standardized associations with HbA1c and cortisol. Each domain-biomarker combination was modeled separately with age, gender, BMI, and lifestyle activity; q values are FDR adjusted across all 14 tests.

Domain	POMP mean (95% CI)	HbA1c β (95% CI), q	Cortisol β (95% CI), q
Self-Inflicted Stress	69.5 (66.9-72.1)	0.273 (0.126-0.420), q<0.001	0.220 (0.084-0.356), q=0.002

Time Management Stress	57.0 (54.8-59.3)	0.338 (0.200-0.477), q<0.001	0.210 (0.076-0.344), q=0.002
Supervisor-Related Stress	55.5 (52.3-58.8)	0.344 (0.189-0.499), q<0.001	0.201 (0.072-0.330), q=0.002
Publication/Outcome Stress	55.2 (52.3-58.2)	0.520 (0.373-0.666), q<0.001	0.211 (0.072-0.351), q=0.003
Evaluation Stress	53.3 (50.4-56.1)	0.361 (0.213-0.509), q<0.001	0.257 (0.143-0.371), q<0.001
Collaboration Stress	50.6 (47.9-53.4)	0.379 (0.242-0.515), q<0.001	0.255 (0.118-0.393), q<0.001
Peer Comparison Stress	50.3 (47.3-53.2)	0.359 (0.209-0.509), q<0.001	0.357 (0.218-0.497), q<0.001
